# Impact of the *PEARLS Healthcare Debriefing* cognitive aid on facilitator cognitive load, workload, and debriefing quality: a pilot study

**DOI:** 10.1186/s41077-022-00236-x

**Published:** 2022-12-12

**Authors:** Michael Meguerdichian, Komal Bajaj, Rachel Ivanhoe, Yiqun Lin, Audrey Sloma, Ariel de Roche, Brian Altonen, Suzanne Bentley, Adam Cheng, Katie Walker

**Affiliations:** 1 Department of Emergency Medicine at NYC Health+Hospitals: Harlem Hospital Center, 506 Malcolm X Blvd, New York, NY 10037 USA; 2 Columbia University School of Medicine, New York, NY USA; 3 NYC Health + Hospitals Simulation Center, Bronx, NY USA; 4 Obstetrics and Gynecology, NYC Health + Hospitals: Jacobi Medical Center, Bronx, NY USA; 5 Albert Einstein College of Medicine, Bronx, NY USA; 6 Department of General Surgery at Rutgers New Jersey Medical School, Newark, NJ USA; 7 University of Calgary, Calgary, Alberta USA; 8 Department of Emergency Medicine at Jacobi Medical Center, Bronx, NY USA; 9 Simulation Coordinator at the Columbia University School of Nursing, New York, NY USA; 10 NYC Health + Hospitals: Research Administration, New York, NY USA; 11 Department of Emergency Medicine, NYC Health + Hospitals: Elmhurst Hospital Center, Queens, NY USA; 12 Simulation Education, NYC Health + Hospitals: Elmhurst Hospital Center, in Queens, NY USA; 13 Mt. Sinai School of Medicine, New York, NY USA; 14 Department of Pediatrics and Emergency Medicine at Alberta Children’s Hospital, Calgary, Alberta USA; 15 University of Calgary, Calgary, Alberta USA; 16 Mater Hospitals, Brisbane, Australia

**Keywords:** Faculty development, Cognitive aid, Debriefing, Cognitive load, Workload, Skill acquisition

## Abstract

**Background:**

The Promoting Excellence and Reflective Learning in Simulation (PEARLS) Healthcare Debriefing Tool is a cognitive aid designed to deploy debriefing in a structured way. The tool has the potential to increase the facilitator’s ability to acquire debriefing skills, by breaking down the complexity of debriefing and thereby improving the quality of a novice facilitator’s debrief. In this pilot study, we aimed to evaluate the impact of the tool on facilitators’ cognitive load, workload, and debriefing quality.

**Methods:**

Fourteen fellows from the New York City Health + Hospitals Simulation Fellowship, novice to the PEARLS Healthcare Debriefing Tool, were randomized to two groups of 7. The intervention group was equipped with the cognitive aid while the control group did not use the tool. Both groups had undergone an 8-h debriefing course. The two groups performed debriefings of 3 videoed simulated events and rated the cognitive load and workload of their experience using the Paas-Merriënboer scale and the raw National Aeronautics and Space Administration task load index (NASA-TLX), respectively. The debriefing performances were then rated using the Debriefing Assessment for Simulation in Healthcare (DASH) for debriefing quality. Measures of cognitive load were measured as Paas-Merriënboer scale and compared using Wilcoxon rank-sum tests. Measures of workload and debriefing quality were analyzed using mixed-effect linear regression models.

**Results:**

Those who used the tool had significantly lower median scores in cognitive load in 2 out of the 3 debriefings (median score with tool vs no tool: scenario A 6 vs 6, *p*=0.1331; scenario B: 5 vs 6, *p*=0.043; and scenario C: 5 vs 7, *p*=0.031). No difference was detected in the tool effectiveness in decreasing composite score of workload demands (mean difference in average NASA-TLX −4.5, 95%CI −16.5 to 7.0, *p*=0.456) or improving composite scores of debriefing qualities (mean difference in DASH 2.4, 95%CI −3.4 to 8.1, *p*=0.436).

**Conclusions:**

The PEARLS Healthcare Debriefing Tool may serve as an educational adjunct for debriefing skill acquisition. The use of a debriefing cognitive aid may decrease the cognitive load of debriefing but did not suggest an impact on the workload or quality of debriefing in novice debriefers. Further research is recommended to study the efficacy of the cognitive aid beyond this pilot; however, the design of this research may serve as a model for future exploration of the quality of debriefing.

## Background

Healthcare debriefing is the process by which performance in a clinical or simulated environment is explored via facilitated conversation [1]. The goal of healthcare debriefing is to reflect on action in order to promote continuous learning [2]. This provides healthcare workers opportunities to gain an understanding of the thoughts and reasons behind their actions in order to replicate, modify, or amend their performance in future events [3]. Several approaches to debriefing have been established [4, 5] as well as courses and publications to support debriefing facilitators [6–8]. Many existing debriefing models are highly structured in nature, thus reducing the facilitators’ ability to adapt the debriefing method to learner needs or learning contexts. A blended approach to debriefing entitled PEARLS (Promoting Excellence and Reflective Learning in Simulation [8] was developed to address this issue. The PEARLS model integrates various debriefing strategies, including learner self-assessment, focused facilitation, and directive feedback, allowing the facilitator to choose from one or more of these strategies. To further support the novice facilitator in applying the PEARLS model, Bajaj et al. developed the PEARLS Healthcare Debriefing Tool as a cognitive aid [9].

The PEARLS Healthcare Debriefing Tool outlines the PEARLS model for debriefing in a table showing its five phases: setting the scene, reactions, description, analysis, and application/summary. The table identifies the objective, the task, and sample phrases for each step and is represented on the front side or the first page of the tool. The analysis phase is expanded on the second page (or reverse side of the page), delineating the key performance domains to consider during a debrief. Additionally, it outlines and scripts the three major educational strategies that constitute the blended approach that defines the PEARLS model [8]. This tool streamlines the model, making it easier for facilitators to access key phrases during a debriefing to support the effective implementation of PEARLS [9].

The use of cognitive aids in technical fields such as aviation and the operating room has resulted in quality and safety improvements and their effectiveness is increasingly a topic of frequent research [10, 11]. Some preliminary studies have explored cognitive aids with respect to satisfaction of participants in debriefings showing little difference [12]. Otherwise, little is known about the impact of cognitive aids on other aspects of debriefing including learning, execution, and quality. The purpose of this pilot study is to characterize what impact, if any, the PEARLS Healthcare Debriefing Tool has with regard to three measurable outcomes: the cognitive load of the facilitator, the workload of the facilitator, and the quality of debriefing performance.

These particular measurements are of interest because they refer to how the cognitive aid may impact the acquisition or execution of debriefing skills. All debriefers are charged with two tasks when performing a debriefing: the learning or further development of debriefing skills and the execution of the debriefing [13]. Cognitive load theory posits that the human cognitive system is limited with respect to its working memory, hampering the ability to process and package large amounts of information. As debriefing is a skill that requires significant development over time, it is considered a complex task [6]. Cognitive load theory mitigation strategies exist to help focus cognitive resources to maximize a learner’s ability to create a long-term memory [14–18]. The workload is determined by the demands of the task including the objectives, timing, structure, number of participants, and complexity of the conversation among other elements [19–21]. Fraser et al. suggest that many of the skills used to mitigate the cognitive load of debriefing may also mitigate some of the workload associated with debriefing [13]. A cognitive aid may reduce that cognitive load as it makes the task of debriefing more manageable allowing cognitive space to learn debriefing skills [13, 22]. If designed well, the aid may lay the foundation for the structure of the conversation leading to an organized debriefing with consistent delivery and reducing the inherent workload of the debriefing.

Cognitive load is distinguished from the quality of the debriefing recognizing that the task has not only been accomplished but accomplished well. The quality of debriefing performance relates to assessing whether or not the debriefer delivers an engaging, organized, and reflective experience in a psychologically safe context [3, 23, 24]. Overall, it can be theorized a useful cognitive aid should foster the effectiveness and caliber of the debriefing.

All these concepts overlap as they are all happening simultaneously. An individual’s cognitive resources for learning, in the case of cognitive load, may be depleted by the increased workload needed to execute a debriefing. Similarly, with such a high cognitive demand on executing this new skill, the quality of the debriefing may be negatively impacted. Ideally, a cognitive aid should positively impact all these elements. By design, it can create a worked example to learn from, promoting the learning of the skill. It can offer scripting to support debriefing execution. It can also offer phraseology in the scripting to promote quality in the debriefing conversation.

To assess a cognitive aid, measurements from different vantage points help to better refine the tool with respect to utility, impact on skill acquisition of the task being performed, and debriefing performance. We hypothesize that utilization of the PEARLS Healthcare Debriefing Tool will result in reduced cognitive load and workload for the facilitator while improving debriefing quality. We approach our hypothesis in this study by measuring the impact of the PEARLS Healthcare Debriefing tool on the perceived cognitive load and workload of the user as well as the quality of their debriefing.

## Methods

### Study design and participants

This experimental pilot study was a double-blinded randomized controlled trial, aimed at characterizing the impact of the PEARLS Healthcare Debriefing Tool on facilitator cognitive load, facilitator workload, and debriefing quality. The study took place at the NYC Health + Hospitals Simulation Center during July and August 2018. The study was approved by the Biomedical Research Alliance of New York (Protocol #FAS-1). We recruited interprofessional fellows from the New York City Health + Hospitals Simulation Fellowship Program and randomly assigned them to 2 groups after attending a 7-h and 1-day “Introduction to Debriefing” course at the start of their fellowship. The population for the study were all novice debriefers and had not received any formalized debriefing training prior to the 1-day course.

### Randomization

Each participant was assigned a number for the purpose of the study. A nurse educator, who was not involved in this research, generated the randomization sequence with an online random number generator. Each participant was randomly assigned to either the intervention group (i.e., receiving the PEARLS Debriefing Tool) or the control group (i.e., not receiving the tool). A total of 7 participants were chosen for each group.

### Intervention

Each participant completed a demographic and experience survey prior to the initiation of this study. Both groups completed a 1-day and 7-h course on how to utilize the PEARLS debriefing model. Participants were introduced to the different phases through a didactic format and a singular experience modeling the phases. Using a trigger video, a collaborative experience was also dedicated to exploring frames. Frames are defined as the set of assumptions on which decisions and actions are based [23]. Following these introductions to debriefing, the participants engaged in collaborative learning sessions of applying the PEARLS debriefing model to trigger video situations. The trigger videos were prepared with specific embedded frames so that fellowship co-faculty could best standardize the practice debriefing sessions. At this stage of the course, the first cohort, the intervention group, had the opportunity to carry out a practice debriefing while utilizing the PEARLS tool in paper form. The control group had the same amount of time to carry out a practice debriefing without the use of the tool. The control group was blinded to the existence of the tool, and the intervention group was instructed to abstain from sharing the tool until after the study was completed. After each debriefing, feedback was offered by the co-facilitators. The fellowship director was the lead instructor of the course. The course agenda can be found in Table 1.

**Table 1:** Introduction to a debriefing course outline

Activity	Description
Introduction to course	• Facilitators and participants become familiar • Psychological safety established
Experiential learning theory didactic	• Explore Kolb’s theory and consider how reflection can promote learning
Interactive simulated debriefing	• Participants experience a trigger video that is debriefed by the facilitator • Participants engage in +/delta reaction to the debriefing
Psychological safety	• Participants explore how to establish and maintain psychological safety
Frames exercise	• Participants explore how their point of view may differ from a learner’s point of view • Participants consider how to approach debriefing conversations aware of their biases
PEARLS framework didactic	• Participants are introduced to the framework provided by Eppich et al. and are exposed to each phase
Debriefing practice	• Participants practice debriefing by applying the framework using trigger videos and simulated actors as debriefing participants • Participants receive feedback on their debriefing with respect to framework, psychological safety, and exploration of frames • Debriefing and feedback happen over 3 cycles

### Simulation scenarios

Following education and practice opportunities, all participants observed one video of a simulated clinical event each week for the following 3 weeks. The participants were then asked to be the lead debriefer of each video scenario. The three different videos depicted three healthcare students in the following scenarios: (A) performing a stretcher-to-stretcher transfer without a transfer board; (B) performing a blood draw on a patient; and lastly (C), a receptionist, nurse, and physician responding to a patient who called to report significant medication side effects. The diversity of the scenarios was intentional to avoid participant context experts having a potential advantage when performing the debriefings. We also chose simulated debriefings, as opposed to real debriefings, so that we could standardize frames and the debriefing situation to better focus attention on the tool. In all three scenarios, there were three characters representing the participants in the debriefing sessions. Each participant watched the video, in isolation prior to the debrief. The videos were in the same order for all participants.

### Debriefing sessions

All participants conducted a debriefing after watching the respective video, 3 weeks consecutively. Three trained actors, recruited from the fellowship faculty, and served as learners during the debriefings. The actors reviewed the trigger videos and were coached using scripted frames for standardization across debriefing experiences. All debriefings were videotaped.

Both groups were trained in PEARLS methodology but were distinguished by the availability of the cognitive aid. The intervention group had the PEARLS cognitive aid available in the form of a clipboard and a paper copy, while the control had a clipboard with a blank piece of paper, working from memory. The learners with the cognitive aid were not given instruction on how to use the cognitive aid but were offered the resource for reference during the debriefing. How they navigated the use of the tool was their own decision. Participants performed all three debriefings under these conditions.

### Rating and outcome measures

Following each of the debriefings, the participants rated their cognitive load and workload scores. Cognitive load was assessed using the Paas & Merriënboer Scale, a 1-to-9-point scale rating mental effort from very low mental effort to very high mental effort associated with learning a task [14, 17, 25]. The tool has been validated in other learning settings as an effective measure of overall cognitive load [17, 25]. The workload of executing the debriefing was evaluated using the NASA-TLX. This validated multi-dimensional scale has been used across multiple professions, to measure workload estimates while performing a task or immediately after task completion [19]. This tool breaks down the effort to perform the task into mental demand, physical demand, temporal demand, performance, effort, and frustration on a 7-point scale. Scores ranging between 0 and 100 are generated to produce an overall workload rating [19, 26]. In some studies, weighting elements of the NASA-TLX have been used to define the focus of the effort to perform a task. In our study to simplify the methodology and make a single step, an unweighted NASA-TLX, also known as the Raw TLX was applied to the performance [26].

Each of the 42 debriefings was filmed and was reviewed by a rater using the Debriefing Assessment in Simulation in Healthcare (DASH) instructor version, long form [3]. The DASH is the most commonly used tool measuring debriefing effectiveness and consists of six items scored on a 7-point Likert scale. Each item addresses important aspects of a debriefing conversation including [1] establishing an engaging learning environment, [2] managing an engaging learning environment, [3] structuring the debriefing in an organized way, [4] provoking an engaging discussion, [5] identifying and exploring performance gaps, and [6] helping trainees achieve or sustain good future performance [3]. Element 1 is not included in the results as this element relates to setting the stage for the learning experience. The raters were blinded to group assignments, as the camera angle precluded raters from seeing the content on the debriefer’s clipboard. One of the raters had previously attended DASH rater training. To achieve inter-rater reliability, two independent DASH raters, who are faculty, were trained using simulated debriefing videos with debriefers of varying proficiency. These raters then reviewed the same videos and reconvened to ensure inter-rater reliability was being maintained. After video rating training, the percentage of agreement increased from 20 to 100% between the raters, the intra-class correlation coefficient (ICC) for agreement with single measures improved from 0.385 (95%CI −0.114, 0.886) to > 0.999. Once achieved, the independent raters evaluated the remainder of the study videos independently. The Pass & Merrienboer Scale, raw TLX, and DASH were performed for all 42 debriefings.

### Statistical analysis

The Paas-Merriënboer scale results between the two groups were analyzed using the Wilcoxon rank-sum test. Mixed-effect linear regression models were conducted to assess the effect of tool impact on debriefing workloads, adjusting for repeated measures in 3 scenarios. Similarly, mixed-effect linear regression models were applied to the effect of the tool on DASH scores, adjusting for repeated measures in the 3 scenarios. In all of these statistical analyses, a *p* value less than 0.05 was considered statistically significant.

As this was a pilot study, a convenience sample was used with an *N* of 7 for each group. The sample size allowed us to detect a large effect size (Cohen’s *d* = 1.6) with a significance level of 0.05 and a power of 0.8. With 3 repeated measures, the number of observations in the mixed-effect linear regression model was 42. Based on the rule of thumb of 10–15 observations for each parameter in the model, we could have a maximum of 3 parameters in the model.

## Results

### Demographics

Participants included both male and female physicians, nurses, and non-clinical administrators representing a broad range of age groups. Five participants reported having some baseline debriefing experience prior to the study, with three of these participants reporting prior use of the PEARLS tool specifically. The demographic and experience data returned by participant surveys are outlined in Table 2. The demographic data demonstrated no statistical differences between groups.

**Table 2:** Demographic characteristics of participants in both groups

		Debrief without tool control group (*n*=7)	Debrief with tool intervention group (*n*=7)
Age	25–34	1 (14.3%)	3 (42.8%)
	35–44	2 (28.6%)	2 (28.6%)
	45–54	2 (28.6%)	2 (28.6%)
	55–64	2 (28.6%)	0 (0%)
Gender	Female	4 (57.2%)	6 (85.7%)
	Male	3 (42.8%)	1 (14.3%)
Profession	MD/DO	3 (42.8%)	6 (85.7%)
	RN/NP	2 (28.6%)	1 (14.3%)
	Non-clinical Administrator	2 (28.6%)	0 (0%)
Percentage of teaching responsibilities in current role (%)	Mean (SD)	15.0 (25.0)	13.9 (17.2)
Participation in simulation (as a trainee) within 3 months	Yes	2 (28.6%)	6 (85.7%)
	No	5 (71.4%)	1 (14.3%)
Currently involved in facilitating simulation	Yes	2 (28.6%)	4 (57.2%)
	No	5 (71.4%)	3 (42.8%)
Previous debriefing training	Yes	3 (42.8%)	2 (28.6%)
	No	4 (57.2%)	5 (71.4%)
Previous experience using the PEARLS tool	Yes	2 (28.6%)	1 (14.3%)
	No	5 (71.4%)	6 (85.7%)

### Facilitator cognitive load

Table 3 displays the median PAAS scores of all three scenarios to assess the mental effort afforded to learn the task of debriefing associated with using the tool. Those who debriefed with the tool had a significantly lower PAAS score than those who did not use the tool in scenario B (effect size *r* = 0.558, *p* = 0.043) and C (effect size *r* = 0.645, *p* = 0.031). No significant differences were detected in scenario A (effect size *r* = 0.421, *p* = 0.1331)

**Table 3:** PAAS score in groups debriefing with and without tool

Median (IQR)	Debrief with tool	Debrief without tool	*p* value
Scenario A	6 (6–6.5)	6 (5–6)	0.1331
Scenario B	5 (5–6)	6 (6–7)	0.043
Scenario C	5 (5–5.75)	7 (6.25–7)	0.031

### Facilitator workload

Table 4 displays the NASA-TLX mean combined scores of all three scenarios debriefed by participants, with and without the tool. Magnitude values for each parameter are on a 100-point scale. The greatest differences between the two groups are seen in mental demand and temporal demand. The calculated mean difference of participants debriefing without the tool rated mental demand required to perform the simulated debriefings 1.6 points higher and temporal demand 2.4 points higher than those debriefing with the tool. Despite the slight increases in cognitive demand noted in these subscale elements without the tool, there was no statistical significance noted between the average scores of the two groups (*p*=0.456). Breaking the tool into its different workload demand elements showed no statistical difference in mental (*p*=0.336), physical (*p*=0.705), temporal (0.302), performance (*p*=0.713), effort (0.928), or frustration (*p*=0.456).

**Table 4:** NASA-TLX scores in groups debriefing with and without tool

Mean (95%CI)	Debriefing with tool (intervention group)	Debriefing without tool (control group)	Mean difference	*p* value
Mental demand	59.5 (48.5, 71.0)	67.5 (56.5, 79.0)	8.0 (−7.5, 24)	0.336
Physical demand	20.5 (9.5, 31.5)	23.5 (12.5, 34.5)	1.8 (−12.5, 18.5)	0.705
Temporal demand	38.5 (23, 53.5)	50.5 (35.0, 65.5)	12 (−9.5, 33.5)	0.302
Performance	46 (37.5, 54.5)	43.5 (35.0, 52.0)	−2.5 (−14.5, 9.5)	0.713
Effort	59.0 (48.0, 70.0)	59.5 (48.0, 70.5)	0.5 (−15.0, 16.0)	0.928
Frustration	41 (28.0, 54.5)	47 (33.5, 60.0)	5.5 (−13.0, 24.0)	0.563
Average score	44.0 (35.5, 52.5)	48.5 (40.0, 57.0)	4.5 (−7.0, 16.5)	0.456

Scores of each domain ranged from 0 to 100

### Debriefing quality

Table 5 details the DASH scores. The debriefers in the study were not given the opportunity to perform this element and thus were not part of the analysis. Elements 4 and 5, referring to provoking an engaging conversation and identifying and exploring learning gaps, showed the largest mean differences at 0.6 and 0.7, respectively. In comparing composite scores, however, there were no significant differences detected in scores between the two groups (*p*=0.436). Debriefers with the tool seem to have slightly lower DASH scores, but the differences were not statistically significant.

**Table 5:** DASH scores in groups debriefing with a tool and without tool

Mean (95%CI)	Debriefing with tool	Debriefing without tool	Mean difference	*p* value
Element 2	4.7 (4.0, 5.5)	5.1 (4.2, 5.9)	0.3 (−0.8, 1.5)	0.582
Element 3	4.9 (4.1, 5.6)	5.2 (4.4, 6.0)	0.3 (−0.7, 1.4)	0.547
Element 4	4.6 (3.8, 5.5)	5.3 (4.4, 6.2)	0.6 (−0.6, 1.9)	0.321
Element 5	4.5 (3.6, 5.4)	5.2 (4.2, 6.2)	0.7 (−0.6, 2.0)	0.322
Element 6	4.9 (4.0, 5.7)	5.2 (4.3, 6.1)	0.3 (−0.9, 1.6)	0.608
Composite score	23.6 (19.8, 27.5)	26.0 (21.7, 30.2)	2.4 (−3.4, 8.1)	0.436

Scores for each element ranged from 1 to 7

## Discussion

Debriefing is a crucial skill that is applied to simulation education as well as after events in the clinical setting [27–29]. In every situation where debriefing is applied, significant skill is needed to navigate conversations and focus on learning objectives. Faculty development, support, and feedback are needed to mature debriefing skills to best adapt to different situations [6, 7, 30, 31]. A cognitive aid that can both support and foster the learning of debriefing could hasten the acquisition of such an important skill.

In this study, data regarding the impact of the PEARLS cognitive aid demonstrates that learning may be improved. In two of the three debriefing scenarios, learners reported that the cognitive load was less when using the tool. In other words, the mental demand required by the working memory to learn debriefing was lessened, suggesting there are more opportunities and mental bandwidth available for learning “how to” debrief. Considering the complexity of debriefing conversations, significant time and effort are required to achieve proficiency. This constitutes preliminary evidence to suggest that the use of the PEARLS tool may impact the efficiency of skill acquisition. This result, as well as all other results, should be appreciated with caution, as the study was underpowered at 0.32. Had the study participants been multiplied by 4, a marked increase in sample size, and a clearer appreciation of statistical significance could be appreciated.

The impact of the cognitive aid with respect to the facilitator workload of debriefing and the quality of the debriefing did not result in a decrease in the cognitive effort to perform the task nor improve the quality of performance. Results associated with the workload of the task were expected to mirror those results appreciated by the PAAS & Merriënboer scale. It is possible that the use of the raw TLX in place of weight NASA-TLX may have impacted the results [26]. By focusing the NASA-TLX through a weighting process, the task would have likely appreciated more mental and temporal demands. Using this weighted scale, the study may have recognized that the cognitive aid would have decreased cognitive workload. It is also important to note that physical demand on this exercise has been included but unlikely adds value to the data as there is little physical effort put into a debriefing conversation. Alternatively, the application of a cognitive aid may have added an unintended cognitive load to the execution of the debriefing because it was unfamiliar and distracted from the user’s performance. Repeat application and familiarization of the tool may have better appreciated the value of the tool. Increased powering through both the number of participants and the number of debriefings within the study might better reflect its impact.

The DASH results demonstrated no specific performance improvement while using the tool. A high-quality debriefing performance often requires a significant amount of practice over an extended period of time and through experiencing a wide range of different settings, to gain crucial skills. It is likely that three debriefings were not enough to appreciate the potential of the tool and more practice with the tool may lend to a difference to be appreciated. The addition of the tool may have added some distracting mental effort because it adds another interactive element to the already complex task of debriefing. For example, when considering the creation of an engaging learning environment, the subtle effective nonverbal gestures that would normally exist may have been overshadowed by looking down at the tool and figuring out how best to use it. That distraction may have been worsened, had the intervention group used a device like a phone or a tablet in place of the paper-based aid. Like any other tool that is being incorporated into a workflow, formal training to achieve familiarity and understanding of how to use the tool will likely precede the skills for using it effectively. Had we continued to keep the two groups randomized over a longer period of time and repeated the measures at more distant time frames, we may have appreciated a significant difference in tool utilization.

This cognitive aid is just one piece in a jigsaw puzzle of skills required to enhance debriefing. Using this tool in a variety of settings with feedback may enhance the tool’s application. Coaching and feedback on debriefing skills provide an opportunity for germane processing of debriefing and motivate change behaviors to impact debriefing skills [13]. Adding feedback and exploring the use of the tool during debriefing experiences may have contributed to a quicker and defter application of the cognitive aid, resulting in higher scores in both the NASA-TLX and DASH. Exploring coaching strategies with and without the tool is another opportunity for further research.

Lastly, it must be considered that the design of the tool may not lend to ease of navigation in performance. Tighter scripting may be one place where the tool may enhance performance in a debriefing as scripted debriefs have proven to be effective [5, 32–34]. Tighter scripting may also create the opposite impact on learning. Although there is learning from performing something well through tight scripting, the opportunity to explore and deviate and make mistakes when performing a debriefing contributes to the growth of debriefing skillsets. Another option with the design may be to consolidate the cognitive aid to a single page to avoid maneuvering from the structured debriefing phases to the analysis phase. Sizing of scripting and coloration may also have contributed to debriefers refraining from using the scripting as the font was too difficult to see or read.

### Limitations

This pilot study was conducted with a small convenience sample to understand the impact of a cognitive aid. The preliminary results indicate that by using a larger more diverse sample and a longer timeline the full impact of the tool may be better understood. Our power calculations indicated cohorts of 28 would more effectively assess if the cognitive aid delivers impact. We recommend further studies that assess the longitudinal impact on skill acquisition and performance of debriefing with the application of cognitive aids such as the PEARLS Debriefing Tool.

Another consideration where there may be limitations are the tools used to study the aid. The DASH was chosen as the tool to measure the quality of the debriefing in our study as it is commonly used for faculty development [35]. The DASH is a behavior-anchored rating scale that may have some limitations in its utility and has only been validated in the context of video debriefing [3]. Dietz et al. note that despite rater training behaviorally anchored rating scales may suffer variance to the timing of observations, individuals being observed, or contexts of the observations [36]. These and other sources of variance difficult to control like previous debriefing experience, difficult conversation experience, emotional intelligence, and creation of perceived psychological safety may have impacted the quality of the data collected, impacting the observed results. By seeking out more validity and reliability testing of the DASH, including comparative testing with other rating tools like the Objective Structured Assessment of Debriefing [37–39], we may more clearly assess debriefing quality and more effectively measure cognitive aid effectiveness. Alternatively, identifying debriefing quality measures that do not rely on behavior-anchored rating scales may prove more reliable.

## Conclusion

In this manuscript, we have considered the usefulness of the PEARLS Healthcare Debriefing Cognitive Aid in terms of its impact on cognitive load, workload, and debriefing quality. Although a pilot study, our findings suggest that the tool does create an opportunity to support faculty development when applying it toward learning debriefing skills by decreasing cognitive load. There was no significant evidence that the tool reduces workload or improves debriefing quality. As this study is a pilot, further research needs to be conducted to look at a larger sample size to truly understand the impact of this tool. We hope that our description of a structured approach to exploring cognitive aids and offers a model for future research for debriefing tools as well as refinement and development of new tools. This manuscript offers another step forward in discovering and refining ways to efficiently train facilitators in engaging in these impactful conversations.

## Data Availability

All data generated or analyzed during this study are included in this published article.

## Abbreviations

PEARLS: Promoting Excellences and Reflective Learning in Simulation
